# THE COURAGE TO EMBRACE OLD AGE

**DOI:** 10.1093/geroni/igz038.1517

**Published:** 2019-11-08

**Authors:** Jim Vandenbosch

**Affiliations:** Terra Nova Films, Chicago, Illinois, United States

## Abstract

Most popular films and television programs reflect, and thereby reinforce, the common and entrenched cultural perception of aging as the “enemy” of vital living. Aging used to be kept at bay in popular films and TV programs through a stereotyping that allowed ridicule and avoidance. Today, such overt negative portrayals have begun to fall out of favor but are being replaced by a subtler form of ageism—that of “super-aging” where older adults who are seen as successfully holding onto their youthful ways are celebrated and held up as models of “successful” aging. This presentation will give an overview of how most popular films and television programs frame the experience of older adulthood, and will illustrate this with clips from such films. Then, in contrast, clips will be presented from films that present a more holistic and balanced view of elderhood.

